# Phenotypic variability in deficiency of the α subunit of succinate‐CoA ligase

**DOI:** 10.1002/jmd2.12018

**Published:** 2019-03-14

**Authors:** Didem Demirbas, David J. Harris, Pamela H. Arn, Xiaoping Huang, Susan E. Waisbren, Irina Anselm, Jordan P. Lerner‐Ellis, Lee‐Jun Wong, Harvey L. Levy, Gerard T. Berry

**Affiliations:** ^1^ Division of Genetics and Genomics, Manton Center for Orphan Disease Research Boston Children's Hospital, Harvard Medical School Boston Massachusetts; ^2^ Department of Pediatrics Nemours Children's Health System Jacksonville Florida; ^3^ Department of Neurology Boston Children's Hospital, Harvard Medical School Boston Massachusetts; ^4^ Baylor College of Medicine Houston Texas

**Keywords:** lactic acidosis, methylmalonic aciduria, mitochondria, succinate‐CoA ligase

## Abstract

Succinyl‐CoA synthetase or succinate‐CoA ligase deficiency can result from biallelic mutations in *SUCLG1* gene that encodes for the alpha subunit of the succinyl‐CoA synthetase. Mutations in this gene were initially associated with fatal infantile lactic acidosis. We describe an individual with a novel biallelic pathogenic mutation in *SUCLG1* with a less severe phenotype dominated by behavioral problems. The mutation was identified to be c.512A>G corresponding to a p.Asn171Ser change in the protein. The liquid chromatography tandem mass spectrometry‐based enzyme activity assay on cultured fibroblasts revealed a markedly reduced activity of succinyl‐CoA synthetase enzyme when both ATP and GTP were substrates, affecting both ADP‐forming and GDP‐forming functions of the enzyme.

## 1 INTRODUCTION

Succinyl‐CoA synthetase (ADP‐forming EC 6.2.1.5 and GDP‐forming EC 6.2.1.4) deficiency (OMIM 611224), due to defects in the alpha subunit of succinate‐CoA ligase (SUCLG1), is a rare autosomal recessive disorder in which coenzyme A cannot be cleaved from succinate, thus limiting the availability of succinate for Krebs cycle function. The resulting combination of lactic acidosis and methylmalonic acidemia is not known to occur in any other metabolic disorder except SUCLG1 and SUCLA2 deficiencies. The first report of SUCLG1 deficiency is that of three siblings from a consanguineous mating, who died in infancy with profound lactic acidosis as well as elevations of urine methylmalonic acid and Krebs cycle intermediates, respiratory chain enzyme deficiency, and mitochondrial DNA depletion.^1^ A later fatality has also been reported in a child who died at 3 years of age.^2^ Further patient reports demonstrate a range of phenotypic expression in SUCLG1 deficiency.^3^ A recent report describes two siblings who died at 29 and 31 months due to a homozygous mutation in the SUCLG1 protein mitochondrial targeting sequence.^4^ The SUCLA2 deficiency has been a relatively less severe disorder characterized by a Leigh‐like encephalomyopathy with psychomotor retardation, poor growth, neurosensory deafness, and dystonia.^5^, ^6^, ^7^, ^8^, ^9^, ^10^ The mean age of death of patients with an *SUCLA2* gene defect was 20 years, whereas it was 20 months for patients with *SUCLG1* gene defects.^11^ A contributing factor may be the addition of liver disease and/or cardiomyopathy in SUCLG1 deficiency.

We report a child who has a novel homozygous mutation in *SUCLG1* and a much less severe phenotype than reported cases. He is 13 years old with moderate disabilities and difficult behavior. There are few genotype‐phenotype relationships reported and only two descriptions of Krebs cycle enzyme activity in patients with *SUCLG1* gene defects.^3^, ^4^, ^11^, ^12^ To further characterize the *SUCLG1* gene defect in our patient, we developed an liquid chromatography tandem mass spectrometry (LC‐MS/MS)‐based enzyme assay to verify that the hypomorphic mutations were indeed pathogenic.^13^

## 2 CASE REPORT

A Chinese boy, the product of a consanguineous mating (first cousins), was seen for evaluation of frequent vomiting, poor tone, poor coordination, and developmental delay. The pregnancy was full‐term and uncomplicated. Labor and delivery were normal at 38 weeks. Birth weight was 2.8 kg and birth length 50.8 cm. He was breastfed. From birth, he was noted to have poor movement and hypotonia as well as frequent vomiting. Despite multiple formula changes, the vomiting persisted and his weight fell from the 50th percentile at birth to below the 5th percentile by 15 months of age. Metabolic evaluation revealed elevated levels of blood lactic acid (7.7 mmol/L; normal <2.2) and urine methylmalonic acid (134 mmol/mol creatinine; normal <5). Urine ethylmalonic, methylcitric, and 3‐hydroxyisovaleric acids were mildly elevated. Plasma C3‐ and C4OH‐acylcarnitines were also mildly increased. A repeat urine organic acid analysis revealed multiple increases including lactate, 3‐hydroxypropionate, 3‐hydroxyisobutyrate, 3‐hydroxyisovalerate, methylmalonic acid, ethylmalonic acid, succinic acid, glyceric acid, and fumaric acid. This was considered to be a nonspecific profile, not clearly indicative of any metabolic disease.

Brain magnetic resonance imaging demonstrated an incidental left hemispheric cyst, and a lactate doublet in the magnetic resonance spectrum of a left basal ganglia voxel. Because of the possibility that he might have vitamin B12 responsive methylmalonic acidemia, hydroxocobalamin was administered but there was no response over 2 weeks. Dietary protein intake was empirically reduced. The plasma methylmalonic acid levels continued to be markedly elevated, ranging from 3100 to 3600 nmol/L (normal 70‐271). Mutation analyses of methylmalonyl‐CoA‐mutase gene and multiple mitochondrial genes in a panel were unrevealing. His cultured skin fibroblasts complemented all mutase and cobalamin deficient cell lines associated with increased plasma methylmalonic acid levels and no identifiable abnormality in the synthesis of adenosylcobalamin (courtesy of Dr David Rosenblatt). However, [^14^C] propionate incorporation into the tricholoroacetate precipitate was slightly reduced. The [^14^C]methyltetrahydrofolate content was not decreased in a complementary macromolecular labeling study. These results were interpreted as ruling out a methylmalonic acidemia due to methylmalonyl‐CoA mutase deficiency, a deficiency in cobalamin (B_12_) metabolites, as well as an intracellular B_12_ transport defect.

At 33 months of age, he was hospitalized for a febrile illness with recurrent vomiting initially treated intravenously; he was then started on gastric feeding. He has had two episodes during which the serum bicarbonate was less than 15 mmol/L. Muscle biopsy revealed reduced activity of complex I and III, as judged by the activity of rotenone sensitive NADH‐cytochrome C reductase (12.9% of the mean) of the electron transport chain, but was otherwise unremarkable (Center for Inherited Disorders of Energy Metabolism [CIDEM], courtesy of Dr. Chuck Hoppel). Mitochondrial DNA content analysis in skeletal muscle was 72% of control. Because of the combination of lactic acidosis, elevated methylmalonic acid levels in plasma and urine, elevated levels of 2‐methylcitrate in serum and urine, as well as elevated levels of propionylcarnitine in plasma, we considered the possibility that the patient may have a deficiency of succinate‐CoA ligase. We subsequently sent genomic DNA for direct sequencing of *SUCLA2* and *SUCLG1* to Baylor Medical Genetics Laboratory.

Over the first decade of his life, the patient's course was largely one of a static encephalopathy without episodes of acute metabolic decompensation. He was clearly handicapped and exhibited explosive behavior that at times could be dangerous for individuals in his vicinity. While there was no overt liver disease, he had a chronic lactic acidosis.

The patient is currently 13 years of age and making little progress. He eats solid food, but fluids are administered through a G‐tube. Developmental assessment was performed at 26, 32, and 43 months using Bayley Scales, and Vineland Adaptive Behavior Scales. The language scores were 62, 62, and 50. The cognitive results were 55, 60, and 65, while the motor result was 52, 52, and 55. He can walk, though he has an unsteady gait and hyperkinesia. He is inattentive, aggressive, and kicks and bites frequently, although he is generally happy. His current laboratory results include the following: plasma lactic acid 4.5 mmol/L (normal range: 0.5‐2.2), elevated serum pyruvate 0.32 mmol/L (normal range: 0.03‐0.11), plasma total carnitine 69.9 μmol/L (normal range: 32‐84), plasma free carnitine 30.9 μmol/L (normal range: 26‐60), plasma propionylcarnitine 6.94 μmol/L (0.00‐0.87), urine methylmalonic acid 85.8 μg/mg creatinine (0.0‐10.0), serum methylmalonic acid 2833 nmol/L (normal range: 73‐271), and lastly serum 2‐methylcitric acid 328 nmol/L (normal range: 60‐228).

## 3 METHODS

### 3.1 Molecular genetic analysis: Sanger Sequencing

*SUCLG1* sequencing was performed in the Baylor Medical Genetics Laboratory by PCR amplification of the coding exons and the immediately flanking intronic sequences of the *SUCLG1* gene located on chromosome 2p11.2, followed by sequencing of the PCR product in both forward and reverse directions using automated fluorescent dideoxy sequencing method. The Genbank NCBI ID NM_0038491 sequence is used as the reference sequence.

### 3.2 Liquid chromatography tandem mass spectrometry analysis of ATP‐ and GTP‐succinyl‐CoA synthetase activities in fibroblasts

#### 3.2.1 Cell culture

The patient was enrolled in the IRB‐approved Manton Center for Orphan Disease Research Center at Boston Children's Hospital. The control fibroblast cell line was between 20 and 26 passages and disease line from the patient was between 10 and 15 passages. The fibroblast cell lines were grown in MEM medium (11090‐081, GIBCO) with 10% fetal bovine serum (100‐106, BenchMark) supplemented with 100 U/mL penicillin and 100 μg/mL streptomycin (15140‐122, GIBCO) in a 37°C incubator with humidified atmosphere of 5% CO_2_. Cell pellets were harvested from 90% to 100% confluent cells by removing the media, rinsing twice with phosphate buffered saline (PBS) and scraping. Upon centrifugation and washing twice with PBS, the cell pellets were stored at −80°C freezer until lysis for enzymatic analyses.

#### 3.2.2 Enzyme assay

Enzyme assays were performed as previously described.^13^ Briefly, the cell pellets were resuspended in PBS, lysed by sonication, and protein concentration in the homogenate was determined after centrifugation at 14 000*g* for 10 minutes at 4°C. An aliquot of the homogenate to yield a final concentration of 1 mg/mL was incubated in 50 mM Tris buffer pH 8.0 (154563, ALDRICH), 5 mM MgCl_2_ (M2670, Sigma), 10 mM D4‐succinate (DLM‐584, Cambridge Isotope Laboratories), 1 mM ATP (A7699, Sigma) or 1 mM GTP (G8877, Sigma), 1 mM CoA (C3144, Sigma), and oligomycin 2 μg/mL (75351, Sigma) in a 37°C water bath for 6 minutes. Following incubation, one volume of reaction mixture was mixed with nine volume mixture of 25 μL of 0.2 M formic acid (695076, Sigma), 10 μL of 50 μM malonyl‐CoA (M4263, Sigma) as internal standard and 100 μL of acetonitrile (A955‐4, Fisher Scientific), and centrifuged at 14 000*g* for 10 minutes. Then a 40 μL supernatant was transferred to an auto‐sampler vial containing 100 μL of acetonitrile/water (8:2 vol:vol) for LC‐MS/MS analysis.

The calibrators were prepared by mixing 25 μL of 0.2 M formic acid, 10 μL of 50 μM malonyl‐CoA, 100 μL of acetonitrile with 15 μL of succinyl‐CoA concentrations of 166.67, 55.56, 18.52, 6.17, 3.09, and 1.54 μM. The LC‐MS/MS analysis were performed using an LC BEH amide column (186004801, Waters) in a Shimadzu HPLC with two LC‐20AD XR pumps and SIL‐20AC XR auto‐sampler and an AB Sciex QTRAP 5500 mass spectrometer in negative‐ion electrospray ionization mode with multiple reaction monitoring scanning. The parameters for LC‐MS/MS are listed in Table 1.

**Table 1:** LC‐MS/MS settings and conditions

Liquid chromatography gradient conditions			
Time (min)	Flow rate (mL/min)	Solvent A%^a^	Solvent B%^a^
0	0.15	70	30
2	0.15	30	70
6.5	0.15	30	70
7.5	0.15	70	30
10	0.15	70	30

Mass spectrometer settings	
Curtain gas	15 psi
Ion spray voltage	−4500 V
Dissolvation temperature	600°C
GS1 and GS2	20 and 40 psi, respectively
Declustering potential	−110 V
Collision energy (CE) for different MRM transitions	
Succinyl‐CoA	866.2 > 408 (CE −55 eV)
D4‐Succinyl‐CoA	870.2 > 408 (CE −55 eV)
Malonyl‐CoA	852.2 > 808 (CE −40 eV)

a Solvent A, 10 mM ammonium formate in acetonitrile/water (90/10, vol/vol); Solvent B, 10 mM ammonium formate in acetonitrile/water (20/80, vol/vol).

### 3.3 Mitochondrial DNA content analysis

Mitochondrial DNA content analysis was performed in Baylor Medical Genetics Laboratory. The mtDNA and nuclear DNA (nDNA) copy numbers are determined by real‐time quantitative PCR using specific primers for the mitochondrial tRNALeu(UUR) gene and the nuclear ß‐2‐microglobulin gene^14^ in duplicate. The control range was for muscle control specimens from over 300 individuals with suspected mitochondrial disease without defects in genes that cause mtDNA depletion. The total amount of mtDNA in DNA samples was calculated from the difference in threshold cycle numbers of nuclear gene and mtDNA.

### 3.4 Pyruvate dehydrogenase complex and electron transport chain complexes analysis

The analyses for pyruvate dehydrogenase complex and electron transport chain complexes in skeletal muscle was performed in the CIDEM Facility. Pyruvate dehydrogenase complex activity was determined by employing thiamine pyrophosphate, NAD and CoA‐dependent decarboxylation of 1‐^14^C‐pyruvate after activation/inactivation (by preincubation with dichloroacetate/fluoride) as previously described.^15^, ^16^

### 3.5 Molecular modeling

Using Swiss‐Protein database Viewer, homology model of human SUCLG1 (UniProtKB—P53597) was visualized based on the crystal structure of porcine protein (2fp4.pdb).

## 4 RESULTS AND DISCUSSION

The clinical history and physical examination, lactic acidosis, and the elevations of methylmalonic acid, propionylcarnitine, 2‐methylcitric acid, and the enzymology data suggested that this patient may have a variant form of SUCLG1 deficiency. In genomic DNA from the patient, a biallelic c.512A>G variant was found in the *SUCLG1* gene. This corresponds to a p.Asn171Ser replacement in the protein (NCBI Reference Sequence: NM_003849.3). Both mother and father were found to be carriers of the c.512A>G variant. This variant was not observed in individuals of European and African American ancestry in the NHLBI Exome Sequencing Project. The p.Asn171Ser variant is reported at an allele frequency of 0.00001 in ExAC database (1 in 121350) and of 0.000004064 (1 allele in 246072 alleles) in the gnomAD database. It is predicted to be probably damaging by Polyphen and deleterious by SIFT using in silico analyses. This variant occurs at a position that is conserved in human, rhesus, mouse, dog, elephant, chicken, and zebrafish (UCSC Genome Browser).

**Figure 1:**
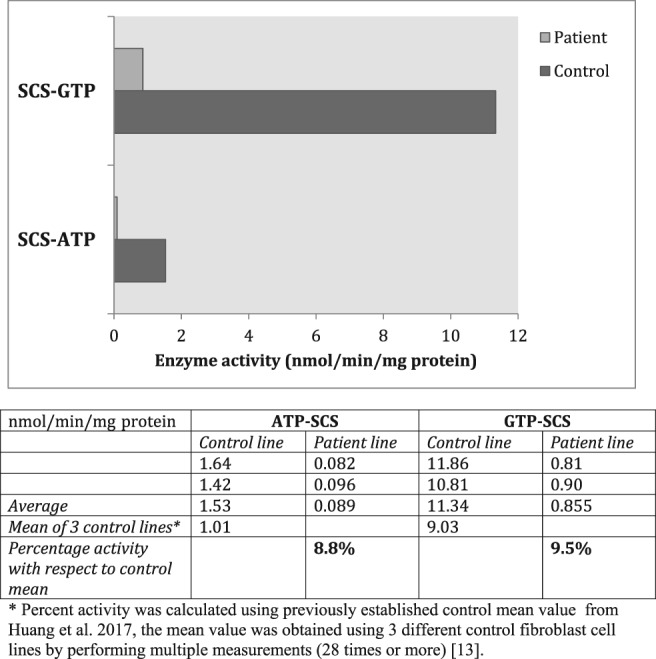
Succinyl‐CoA synthetase activity of cultured patient fibroblasts and control fibroblasts is shown using ATP or GTP as substrate. The activity of the enzyme in patient fibroblasts was 8.8% of control when ATP is the substrate, 9.5% of the control when GTP is the substrate

The *SUCLG1* gene encodes for the α‐subunit of succinate‐CoA ligase (succinyl‐CoA synthetase). As shown in Figure 1, the activity of succinyl‐CoA synthetase enzyme using ATP or GTP as substrate in fibroblasts from the patient revealed a markedly reduced activity when both ATP and GTP were substrates. The activity of the enzyme was 8.8% of control when ATP was the substrate and 9.5% of control when GTP was the substrate. The patient has a phenotype that is milder than the infantile fatal phenotype of the original siblings reported with SUCLG1 disorder,^1^ but also milder than the child in the second report who died just before 3 years of age.^2^ This is not surprising as most genetic disorders are heterogeneous, and even more variability is to be expected. The major problems have been in behavior and language development.

Succinate‐CoA ligase is an enzyme in the citric acid cycle; it plays several important roles such as catalyzing the conversion of succinyl‐CoA to succinate, allows for substrate level synthesis of a nucleoside triphosphate, either ATP or GTP, and is linked to the synthesis of deoxynucleotide triphosphates. Both ATP and GTP are required for the synthesis of essential molecules in the mitochondrial matrix and either may play a role in the synthesis of deoxynucleotide triphosphates in nondividing cells as succinate‐CoA ligase is in complex with the nucleoside diphosphate kinase (NDPK) and GABA transaminase (ABAT).^17^, ^18^ Catalyzing the exchange of phosphate between tri‐ and diphosphoribonucleosides it operates the essential dNTP salvage pathway during mtDNA synthesis.^19^ Succinate‐CoA ligase plays a role in the maintenance of the mitochondrial DNA pool through action of NDPK. We do not understand why the patient did not develop mitochondrial DNA depletion. This is a very important conundrum and whether other more severe mutations in SUCLG1 affect its interaction with NDPK more severely than the p.Asn171Ser mutation requires further study. The succinate‐CoA ligase enzyme also serves at the junction of the catabolism of amino acids and lipids for energy production. It is universally distributed, being present as two isoforms in mammals, one utilizing ADP and the other GDP.^20^, ^21^ The α chains of each are identical, with the specificity residing in the variant β forms. In the mouse embryo, all of the subunits are expressed in muscle, nervous system, kidney, liver, gastrointestinal tract, adrenal and salivary glands, with lower expression of the GDP forming β unit in most of the nervous system.^22^

Individuals with mutations of either the α‐chain (*SUCLG1*), or of the ADP utilizing β chain (*SUCLA2*) have been discovered. There is as yet no reported incidence of a disorder involving the GDP utilizing β‐chain (*SUCLG2*). The biochemical phenotype includes methylmalonic acidemia and lactic acidosis; the latter may be, at least in part, a result of mitochondrial DNA depletion.^23^, ^24^ Yet that was not to be found in the subject of this report, nor in two siblings with *SUCLA2* gene defects that we previously studied.^13^ These children, including our patient, have abnormal metabolites associated with the disorders, propionic and methylmalonic acidemias; these pathway metabolites may inhibit other mitochondrial enzymes essential for energy metabolism.^24^ This might also be one of the reasons for having reduced activity of complex I and III in a muscle biopsy sample when mtDNA depletion is absent.

**Figure 2:**
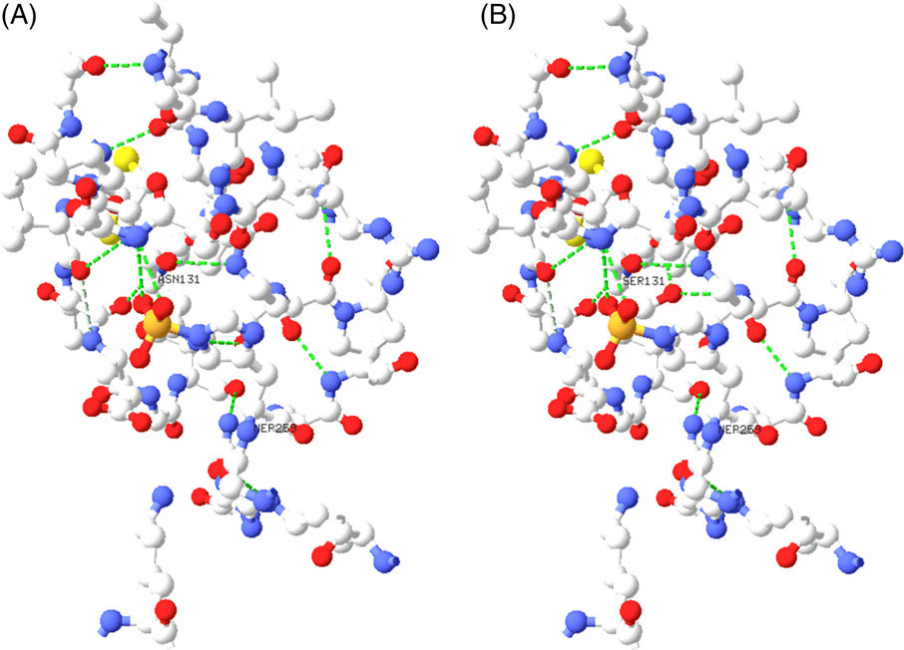
Views of the α‐chain of porcine SUCLG1 within 8 Å of the active site histidine 259: (A) shows the normal wild‐type asparagine at amino acid 131, while (B) shows the serine mutation. Hydrogen bonding is indicated by the dashed green lines. The human N171 corresponds to pig N131, and H299 at the active site of human protein corresponds to H259 in pig protein. The homology between the human and porcine proteins is 95.7%

While functional studies of the p.Asn171Ser were not performed, the marked reduction in enzyme activity with both substrates confirms the diagnosis and supports pathogenicity for the c.512A>G variant. Also, the site of the residue is highly conserved and appears to be facing the active site (Figure 2). Interestingly, the c.509C>G variant causing a change in the neighboring amino acid from proline to arginine at location 170 (p.Pro170Arg) was reported in a case of SUCLG1 deficiency with severe antenatal manifestations.^25^

It has become clear that the presence of lactic acidosis and methylmalonic acidemia may indicate mutations in either of the two forms of succinyl‐CoA synthetase: the ADP or GDP binding.^23^ The former appears to be associated with tissues that are more catabolically active, while the latter is associated with anabolic reactions. It also functions in the regulation of ATP production through the binding of phosphate.^26^ We do not know the details of the pathway from the mutation to the phenotype, but it is conceivable that in addition to toxic metabolites and impaired energetics, there may be other mechanisms that lead to cell dysfunction.

## AUTHOR CONTRIBUTIONS

*Irina Anselm*: Cared for the patient and helped with the construction of the manuscript.

*Pamela H. Arn*: Cared for the patient and helped with the construction of the manuscript.

*Gerard T. Berry*: Cared for the patient, was overall responsible for the genesis of the LC‐MS/MS enzyme assay and for overseeing the construction of the manuscript.

*Didem Demirbas*: Performed laboratory work and wrote the manuscript.

*David J. Harris*: Cared for the patient and wrote the manuscript.

*Xiaoping Huang*: Performed the laboratory work and helped with the construction of the manuscript.

*Jordan P. Lerner‐Ellis*: Assisted with the laboratory testing and interrogation of the gene mutations.

*Harvey L. Levy*: Cared for the patient and helped with the construction of the manuscript.

*Susan E. Waisbren*: Performed neuropsychology testing on the patient and helped with construction of the manuscript.

*Lee‐Jun Wong*: Performed the mtDNA depletion analysis and the evaluation of the gene mutation and helped with the construction of the manuscript.

## FUNDING INFORMATION

The authors confirm independence from the sponsors; the content of the article has not been influenced by the sponsors.

## ETHICS APPROVAL

All procedures followed were in accordance with the ethical standards of the responsible committee on human experimentation (institutional and national) and with the Helsinki Declaration of 1975, as revised in 2000. This article does not contain any studies with laboratory animals performed by the any of the authors.

## PATIENT CONSENT

Informed consent was obtained from all subjects for being included in the study. The patient skin fibroblasts and patient consent were acquired through an IRB‐approved protocol by Manton Center for Orphan Disease Research at Boston Children's Hospital.
